# Increase of Elderly Population in the Rainstorm Hazard Areas of China

**DOI:** 10.3390/ijerph14090963

**Published:** 2017-08-26

**Authors:** Pujun Liang, Wei Xu, Yunjia Ma, Xiujuan Zhao, Lianjie Qin

**Affiliations:** 1 State Key Laboratory of Earth Surface Processes and Resource Ecology, Beijing Normal University, Beijing 100875, China; liangpujun@mail.bnu.edu.cn (P.L.); mayj@mail.bnu.edu.cn (Y.M.); xjzhao@mail.bnu.edu.cn (X.Z.); qinlianjie@mail.bnu.edu.cn (L.Q.); 2 Academy of Disaster Reduction and Emergency Management, Ministry of Civil Affairs and Ministry of Education, Beijing 100875, China; 3 Faculty of Geographical Science, Beijing Normal University, Beijing 100875, China

**Keywords:** natural disaster risk, rainstorm, tendency, population exposure, China

## Abstract

In light of global warming, increased extreme precipitation events have enlarged the population exposed to floods to some extent. Extreme precipitation risk assessments are of great significance in China and allow for the response to climate change and mitigation of risks to the population. China is one of the countries most influenced by climate change and has unique national population conditions. The influence of extreme precipitation depends on the degree of exposure and vulnerability of the population. Accurate assessments of the population exposed to rising rainstorm trends are crucial to mapping extreme precipitation risks. Studying the population exposed to rainstorm hazard areas (RSHA) at the microscale is extremely urgent, due to the local characteristics of extreme precipitation events and regional diversity of the population. The spatial distribution of population density was mapped based on the national population census data from China in 1990, 2000 and 2010. RSHA were also identified using precipitation data from 1975 to 2015 in China, and the rainstorm tendency values were mapped using GIS in this paper. The spatial characteristics of the rainstorm tendencies were then analyzed. Finally, changes in the population in the RSHA are discussed. The results show that the extreme precipitation trends are increasing in southeastern China. From 1990 to 2010, the population in RSHA increased by 110 million, at a rate of 14.6%. The elderly in the region increased by 38 million at a rate of 86.4%. Studying the size of the population exposed to rainstorm hazards at the county scale can provide scientific evidence for developing disaster prevention and mitigation strategies from the bottom up.

## 1. Introduction

Climate change is a global problem that has generated major concern from the global society. The global hydrological cycle has changed as a result of increased water vapor in the atmosphere, which has been induced by increased heating, and has produced more intense precipitation events [1]. The IPCC SREX [2] noted that there have been statistically significant increases in the number of heavy precipitation events (e.g., 95th percentile) since 1950 in some regions. The synthesis report of the fifth assessment of the IPCC [3] noted that in the context of global climate change, the frequency and intensity of future global heavy rainfall events will change to varying degrees, and heavy rainfall events are likely to increase in most parts of the world by the end of the 21st century. This change means that the risk of flooding and waterlogging induced by extreme precipitation events will increase [4]. Flooding is the most frequent and serious natural disaster that affects humans. The global annual losses and causalities caused by flooding constitute approximately 40% to 50% of the total losses caused by natural disasters [5]. On the other hand, economic losses from weather- and climate-related disasters have increased, but with large spatial and interannual variabilities [2]. Economic losses are higher in developed countries, as opposed to the higher fatalities in developing countries, due to the differences in exposure and vulnerability [2]. Hence, exploring the exposure and vulnerability is necessary for disaster loss and risk assessments.

China is seriously affected by meteorological disaster due to its geographical location and climatic conditions [6]. For example, rainstorm-induced floods in China in 1991 caused more than 2000 deaths and 49,973 injuries [7]. Since the 1950s, the frequency of extreme precipitation events in China has increased with regional differences. The middle and lower reaches of the Yangtze River and the southeast coastal areas have had significantly increasing trends [8]. Moreover, the future extreme precipitation events in China are predicted to increase in both the RCP4.5 and RCP8.5 scenarios [9]. In addition, the population in these areas has become denser due to rapid economic growth and urbanization. All of these changes result in more of the population being exposed to rainstorm hazard [10]. The risks of climatic disasters such as high temperatures, flooding and droughts will increase by the end of the 21st century. Urbanization, aging and accumulative wealth will further amplify such risks [11]. Therefore, analyzing the characteristics of the population exposed to RSHA could help create a risk reduction policy for rainstorm-related disasters.

Exposure is one of the main factors that determines the risk of a disaster, and the changing trends in exposure and vulnerability have different impacts on risk [12]. Exposure is used to indicate the number or value of the elements exposed to a hazard [13]. Exposure is dynamic and varies across temporal and spatial scales and depends on economic, social, geographic, demographic, cultural, institutional, governance and environmental factors [14]. Population distribution, growth and changes will affect the extent of population exposure. Previous research on population exposure to natural disasters has mainly focused on the total population exposure [15,16,17], the exposure of different populations [18,19] and the exposure at different times [20,21,22]. Some researchers have studied population exposure by calculating the number of exposed people and the total population density of a hazard area [16,17]. Other researchers have combined a variety of hazard indices with the population index to build a population exposure index [20]. Some studies have also found that vulnerable and diseased people in flooded areas are more susceptible to natural disaster risks, including the elderly, immunocompromised or individuals who speak limited English and have a low socioeconomic status [23,24]. For example, almost half of Hurricane Katrina’s deaths were individuals over 75 years old [25]. Further, after a super-cyclone hit Odisha, India, significantly more children in the high exposure area were found to have post-traumatic stress disorder (PTSD) compared to those in the low exposure area [26]. Older adults, particularly those with poor health, are more vulnerable to the physical effects of disasters [27,28], while they may be more resilient than young adults to the psychological effects of disaster [29]. Extreme precipitation impacts the elderly who are particularly sensitive to climate change that can occur before, during, and after an extreme precipitation event, since the elderly may be involved in activities that put them at risk [30]. Such impacts can be physical, mental, or emotional, including water-related illness, casualties, stress, and economic hardship. The elderly may also have mobility constraints, and the need to evacuate an area can pose increased health and safety risks for them [30]. Therefore, Wood et al. [19] considered the exposure of different parts of the population (residents over 65 years of age, workers, etc.) in a study on population exposure to tsunamis and considered it a variable in the tsunami population evacuation model. Jochem et al. [21] estimated the hourly population densities of airports and cruise port terminals using near-real-time flight data and cruise ship data obtained from the Internet to increase the accuracy of the population exposure estimates and provide additional reference information to emergency responders. Jones et al. [22] used climate scenarios and socioeconomic data to study the future population exposure to heat extremes in the U.S. Several achievements have been made in population exposure studies; however, there have not been any breakthroughs in studies on the population exposure and vulnerability to rainstorm-related disasters. This lack of progress is because standard and unified hazard areas of rainstorm-induced flooding and waterlogging have not been identified. Previous researchers have studied the population exposure from different demographic perspectives and determined the differences of the exposed demographics in the ability to receive and understand disaster warning information [18,19]. China is the most populous country in the world and accounts for one-fifth of the world’s total population [31]. However, there are very few studies on the population exposure of the different vulnerable groups in China.

The population of China is aging faster than that in developed countries [32]. In addition, the population structure, along with urbanization and socio-economic changes, have affected the trend of exposure to extreme climate events [33]. This paper explores the level of population exposure in RSRA and analyzes the changes of the total population and the vulnerable population, as indicated by the proportions of the elderly and children using the population census data at the county level from 1990, 2000 and 2010 in China.

## 2. Data and Methods 

### 2.1. Data

The data used in this study included precipitation and population data. The precipitation data from a total of 756 stations were derived from the historical observation data provided by the China Meteorological Data Service Center. The dataset used in this study was from 1 January 1975 to 31 December 2015. The stations that were missing more than one week of data in a given year were not included in the calculations of this year. The stations that were missing data for more than 10% of the years, or that did not record rainstorms in more than 3 years, were removed. A total of 519 weather stations were used to calculate the rainstorm tendency values.

The population data used in this paper were derived from the national population census data of Mainland China, namely, the fourth census in 1990, the fifth census in 2000 and the sixth census in 2010. The data included the total population, the number of children (age ≤14 years), and the number of elderly (age ≥65 years) at the county level. Taiwan, Hong Kong and Macao were excluded from this study due to a lack of data.

### 2.2. Methods

**Figure 1:**
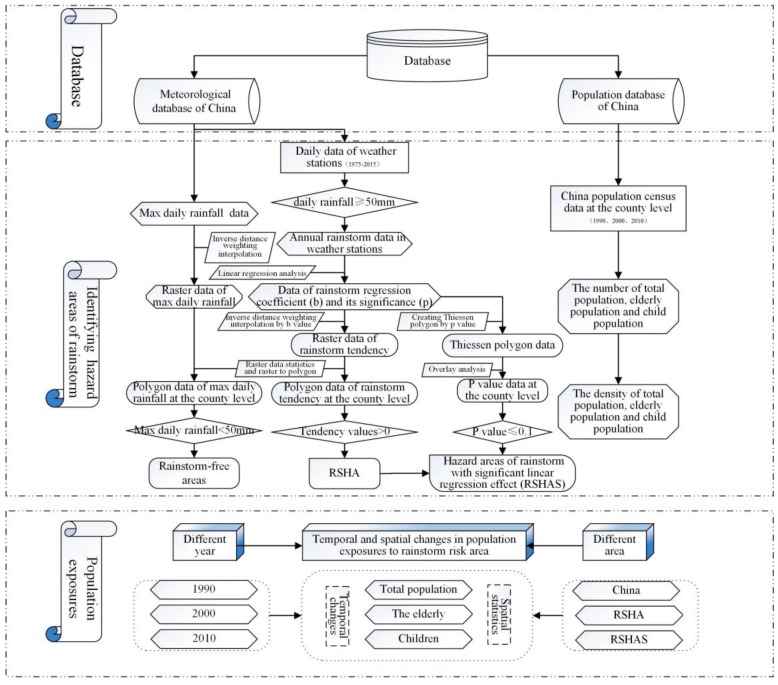
The technical roadmap. RSHA: rainstorm risk hazard area.

The main tasks of this study include two parts, namely, the identification of the rainstorm hotspots or hazard areas and the analysis of the population exposed to the hazard areas. The flow diagram is shown in Figure 1.

#### 2.2.1. RSHA Identification

A set of climate change indices that focus on extreme events were defined by the Expert Team on Climate Change Detection, and these indices have been widely used to analyze global changes in extremes [34,35,36]. Indices of duration, timing, frequency, precipitation amounts, continuity, concentricity, preconditioning, and intensity are usually used to study extreme precipitation changes. For example, Alexander et al. [37] studied the global observed changes in daily precipitation extremes by analyzing the changes in precipitation percentile indices including very wet and extremely wet days. Extreme precipitation events have also been analyzed using the precipitation concentration index, precipitation concentration degree, precipitation concentration period and other indices [38,39]. This paper divides the RSHA according to the trends in the annual precipitation.

A rainfall event totaling 50 mm or more in 24 h is regarded as a rainstorm, according to the China Meteorological Administration [40]. This study identifies the RSHA using the daily precipitation data from weather stations. First, the maximum daily rainfall raster data from all stations from 1975 to 2015 are interpolated using the inverse distance weighted (IDW) method to fill the missing data gaps. Then, if more than 30% of a county has daily precipitation values less than 50 mm after interpolation, the county will be regarded as a rainstorm-free area. Next, the rainstorm regression coefficient and significance for all weather stations are obtained using a linear regression equation (Equation (2)). Then, the spatial distribution of the rainstorm trends in China is obtained using the IDW interpolation method based on the rainstorm tendency values of all stations. The rainstorm tendency value for each county is calculated as the mean value of all grids. Further, Thiessen polygons are built for all weather stations to identify the linear regression effect of the county. Finally, counties with tendency values larger than 0 are defined as rainstorm risk hazard areas (RSHA), and the RSHA with significant linear regression effects are defined as the hazard areas of rainstorm with signification linear regression effect (RSHAS) in this study.

In addition, according to the standard deviations of the rainstorm tendency values, the counties were divided into three types. The average of the tendency values is 0.39 mm/a. The counties with tendency values larger than 4 mm/a (>1.5 Std. Dev) were classified as type I, the counties with values between 1.6 and 4 mm/a (0.5–1.5 Std. Dev) were classified as type II, and the counties with values between 0 and 1.6 mm/a (−0.5–0.5 Std. Dev) were classified as type III.

The calculation methods for the annual rainstorm precipitation, rainstorm tendency values and IDW interpolation are as follows:

The annual rainstorm precipitation *R_ij_* in year *j* for weather station *i* can be calculated by Equation (1):

(1) $$R_{ij}=\sum_{k=1}^{n}r_{ijk}$$

where *n* represents the recorded number of rainstorms for station *i* in year *j* and *r_ijk_* represents the precipitation of the *k*th rainstorm.

The annual rainstorm sequence is *x_i_* for weather station *i*, and the corresponding years are expressed by *t_i_*. The linear regression equation between *x_i_* and *t_i_* is established by Equation (2):

(2) $$x_{i}=a+bt_{i}$$

where *a* is the regression constant and *b* is the regression coefficient; they can be solved using the least squares method. Coefficient *b* indicates the linear trend of the variable. A value of *b* > 0 indicates an increasing trend of *x* with time, and *b* < 0 indicates a decreasing trend of *x* with time. The absolute value of *b* indicates the degree of ascension or descent. In this study, *b* is called the rainstorm tendency value.

Based on the rainstorm tendency values at the weather stations, the predicted rainstorm tendency values (*B*) for the grids of the study area are obtained using IDW interpolation by Equation (3):

(3) B=Σ1mbidipΣ1m1dip

where *b_i_* is the rainstorm tendency value at weather station *i*, *d_i_* is the distance between weather station *i* and the predicted point, *m* is the number of weather stations around the predicted point and *p* is used to control the weight value.

#### 2.2.2. Population Exposure Analysis

The population exposures in the RSHA were derived based on the total population, the number of elderly people and the number of children in 1990, 2000 and 2010 at the county level. Spatially, the number and percentage of the total population, the elderly and children in different regions in 1990, 2000 and 2010 were analyzed. Density maps of the total population, the elderly and children in 1990, 2000 and 2010 were compiled. Temporally, the population growth rate and the total population, the number of elderly, and children in different years and different regions were analyzed. Maps of the change in density of the total population, the elderly and children in 1990–2010, 1990–2000 and 2000–2010 were developed.

## 3. Results

### 3.1. Features of RSHA

**Figure 2:**
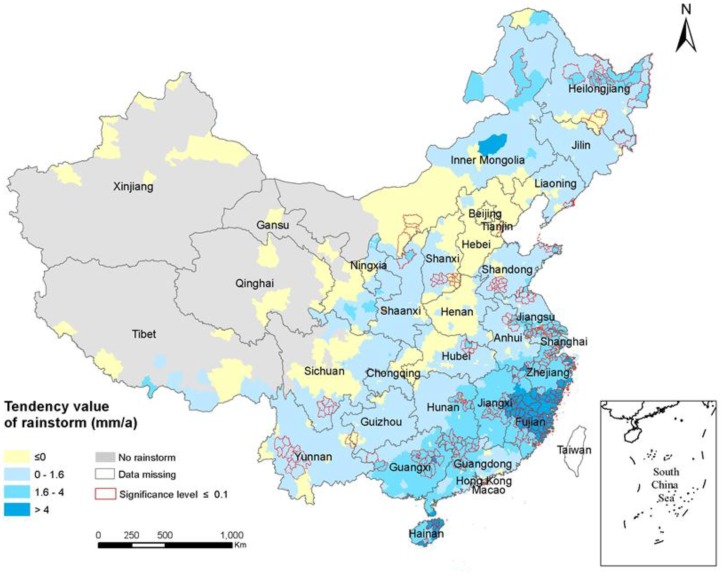
Trend of annual rainstorms from 1975 to 2015.

There are 320 weather stations out of the 519 stations with tendency values greater than 0. There are 1781 counties with tendency values greater than 0, which correspond to the counties where the number of annual rainstorms has increased. The total RSHA is approximately 5.01 × 10^6^ km^2^, which accounts for 52% of China’s mainland area (Figure 2). There are 98 type I counties in the RSHA with a total area of approximately 1.7 × 10^5^ km^2^, which accounts for approximately 1.8% of China’s mainland area. There are 480 type II counties in the RSHA with a total area of approximately 1.24 × 10^6^ km^2^, which accounts for 12.9% of China’s mainland area. There are 1203 type III counties in the RSHA with a total area of approximately 3.6 × 10^6^ km^2^, which accounts for 37.5% of China’s mainland area. As shown in Figure 2, the annual rainfall showed an increasing trend in the southeast and most other areas. There were significant increasing rainfall trends along the southeast coast of Jiangsu, Shanghai, Zhejiang, and Fujian provinces and decreasing trends in Sichuan, Henan, Hebei, and Inner Mongolia.

The linear regression effects of 69 of the 519 weather stations were significant (i.e., passed the significance test). As shown in Figure 2, the counties with red borders were significant. The RSHA with significant linear regression effects were defined as RSHAS in this study. There were 302 counties in the RSHAS. The total area of the RSHAS is approximately 9.6 × 10^5^ km^2^, which accounts for 10% of China’s land area. There are 76 type I counties in the RSHAS with a total area of approximately 1.1 × 10^5^ km^2^, which accounts for 1.1% of China’s mainland area. There are 157 type II counties in the RSHAS with a total area of approximately 5.7 × 10^5^ km^2^, which accounts for approximately 5.9% of China’s mainland area. There are 69 type III counties in the RSHAS with a total area of approximately 2.8 × 10^5^ km^2^, which accounts for approximately 2.9% of China’s mainland area. Most of the counties in the RSHAS were in Fujian, Zhejiang, Jiangsu, Guangdong, Guangxi, Jiangxi and other southeast regions.

### 3.2. Analysis of Population Exposure in the RSHA 

#### 3.2.1. Analysis of Population Exposure

To analyze the population exposed to rainfall, the number and percentage of the total population, the elderly and the children in different regions in 1990, 2000 and 2010 were statistically analyzed (Table 1). The percentages of the elderly and the children of the total population in different regions were also calculated (Table 2).

There were approximately 910 million people (67.9% of the total national population) living in the RSHA in 2010. The type I counties in the RSHA had approximately 47 million people, which accounted for approximately 3.5% of the total national population. There were approximately 83 million elderly people in the type I counties, which accounted for 69.5% of the national elderly population and 9.1% of the total RSHA population, which is close to the national elderly population percentage (8.9%). There were approximately 156 million children in the type I counties, which accounted for 70% of the national child population and 17.1% of the total RSHA population, which is slightly higher than the national child population percentage (16.6%). There were approximately 148 million people living in the RSHAS in 2010, which accounted for 11% of the total national population. There were approximately 36 million people in the type I counties in the RSHAS, which accounted for approximately 2.7% of the total national population. There were approximately 13 million elderly people in the type I counties in the RSHAS, which accounted for 11% of the national elderly population 8.9% of the total RSHAS population, which is similar to the national elderly population percentage (8.9%). There were approximately 24 million children in the type I counties in the RSHAS, which accounted for 10.7% of the national child populations and 16% of the total RSHAS population, which is close to the national child population percentage (16.6%).

The patterns of population exposure in 1990 and 2000 are similar to that in 2010. There were approximately 800 million people (70.6% of the total national population) living in the RSHA in 1990. There were approximately 44 million elderly people in the RSHA, which accounted for 70.3% of the national elderly population and 5.6% of the total RSHA population. There were approximately 226 million children, which accounted for 71.9% of the national child population and 28.2% of the total RSHA population.

In terms of the level of the population exposed in 2000, there were approximately 866 million people (68.4% of the total national population) living in the RSHA. There were approximately 62 million elderly people, which accounted for 70.3% of the national elderly population and 7.2% of the total RSHA population. There were approximately 204 million children, which accounted for 70.3% of the national child population and 23.5% of the total RSHA population.

The RSHA areas cover approximately 52% of the total Chinese mainland. However, the proportions of the total population, the elderly and the children living in the RSHA were all more than 65%, indicating that the majority of the total population, the elderly and the children lived in the RSHA. Moreover, the proportions of the elderly and children in the RSHA and RSHAS were close to those of the national average.

**Figure 3:**
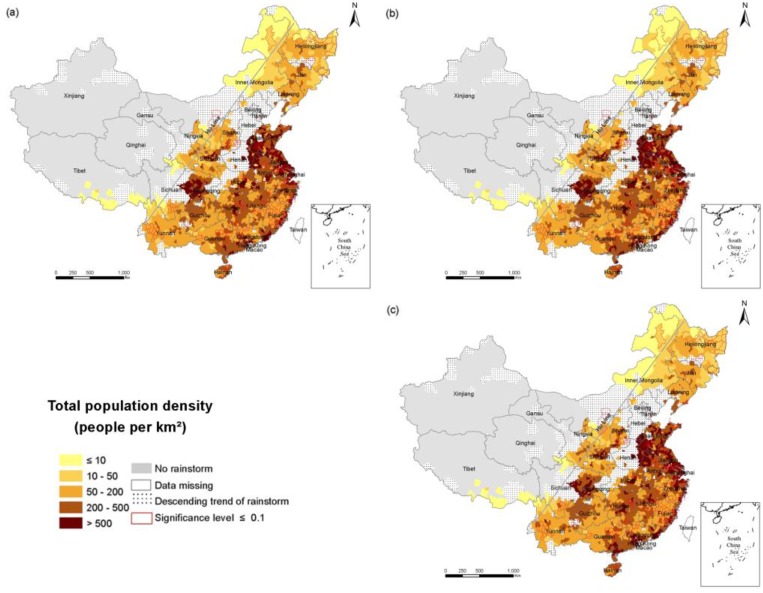
The density of the total population in the RSHA. (**a**) 1990; (**b**) 2000; (**c**) 2010;

**Figure 4:**
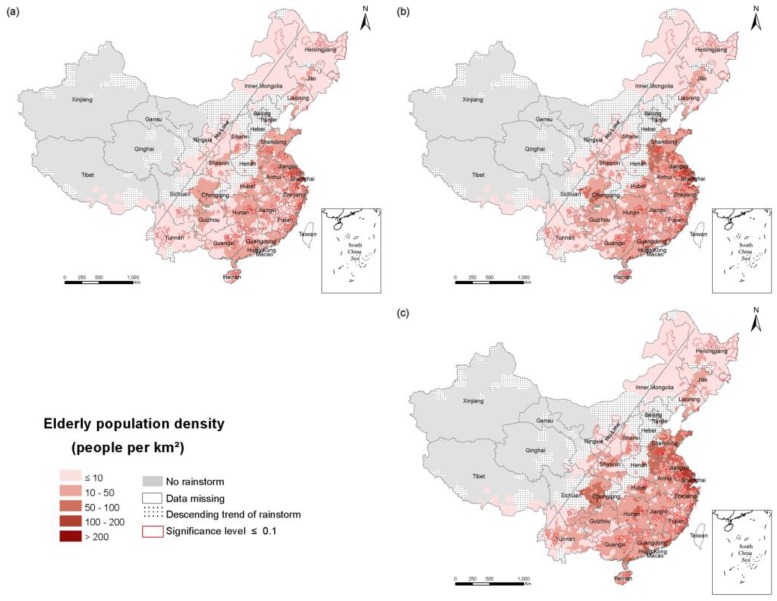
The density of the elderly population in the RSHA. (**a**) 1990; (**b**) 2000; (**c**) 2010.

**Figure 5:**
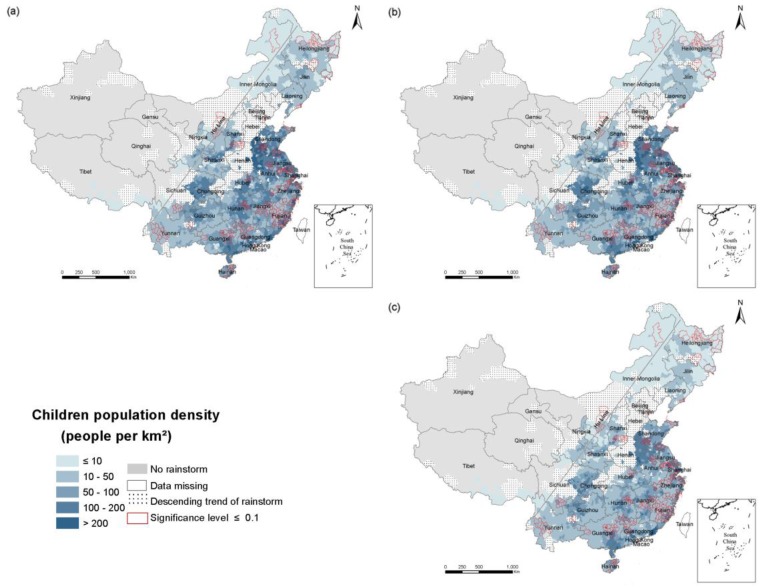
The density of child population in the RSHA. (**a**) 1990; (**b**) 2000; (**c**) 2010.

To explore the spatial and temporal changes of population exposure in the RSHA, density maps of the total population (Figure 3), the elderly (Figure 4) and children (Figure 5) in 1990, 2000 and 2010 were compiled. China’s population density is bounded by the Hu Huanyong line (Hu Line) and more of the population lives in the southeast and less lives in the northwest. Coincidentally, a considerable portion of the RSHA is also distributed east of the Hu Line. In 2010, the highest total population density in the RSHA was concentrated in Shandong-Anhui circle, the Yangtze River Delta (Shanghai, Jiangsu and Zhejiang), Guangdong, Hunan and Sichuan. The counties with a total population density of more than 500 persons/km^2^ were mainly concentrated in central China.

The population density of the elderly in most areas of the south was within the range of 10–50 persons/km^2^, and the counties an elderly population density of more than 50 persons/km^2^ were mainly concentrated in Shandong, Anhui, Jiangsu, Shanghai, Zhejiang, Sichuan, and Chongqing. In the northern region, such as the northeastern part of Inner Mongolia, the elderly population density in the RSHA was mostly less than 10 persons/km^2^. The counties in the RSHA with a density of children greater than 100 persons/km^2^ were concentrated in the Shandong, Anhui, Yangtze River Delta, Guangxi and Guangdong regions.

#### 3.2.2. Analysis of the Change in Population Exposure

Over the past 30 years, China’s total population has increased overall, and the patterns of the population changes in the RSHA and RSHAS were consistent with the overall trend in China. In general, the total population and the number of elderly people increased, and the number of children decreased. To analyze the changes in the population exposure, the population growth rate and the total population, the number of elderly and children in different years and different regions were investigated (Table 3) in this paper.

From 1990 to 2010, the total population increased by approximately 206 million people with an increase rate of 18.2%. The elderly increased by approximately 56 million, which accounted for approximately 27% of China’s population increase. The total population in the RSHA increased by approximately 110 million people with a rate of 13.8%, which is lower than the national population increase rate (18.2%). The elderly in the RSHA increased by 38 million people with a rate of 86.1%, which is slightly lower than the national population increase rate (88.2%). The contribution of the elderly to the total population increase in the RSHA was approximately 34.7%. The number of children decreased by approximately 70 million people at a rate of 31%, which is less than the national child population decrease rate (29%). The absolute value of the child population rate of change was less than the absolute value of the elderly population rate of change in the RSHA.

The total population of type I counties in the RSHA increased by approximately 10 million people with a rate of 28.6%, which is much higher than the total population growth rates (19.8% and 10%) of the types II and III counties and higher than that of RSHA overall and the national level (13.8% and 18.2%). The elderly population of type I counties increased by 1.7 million with an 84.4% growth rate, and the contribution of the elderly to the total population in the type I counties was approximately 16%. The number of children in the type I counties decreased by 3.9 million with a change rate of −34.7%.

The regularity of the population exposure change in the RSHAS is similar to that in the RSHA. The total population in the RSHAS increased by 20.6% between 1990 and 2010 with an absolute increase of 25 million, which was higher than total population growth rates in both the RSHA and China (13.8% and 18.2%). The elderly in the RSHAS increased by 84.5% with an absolute increase of 6 million, which contributed to approximately 24% of the total population increase in the RSHAS. The number of children decreased by 10.5 million with a change rate of −30.6%. The total population of the type I counties in the RSHAS increased by 8.8 million with a 32.2% growth rate, which was higher than the growth rates of the total population of types II and III counties (20% and 11%). This growth rate also exceeded the total population growth rates in the RSHA and China (20.6% and 18.2%). The elderly of type I counties in the RSHAS increased by 1.3 million with an 87.4% growth rate, which contributed to approximately 14.8% of the total population increase of type I counties in the RSHAS. Compared with 1990, the number of children in the type I counties in 2010 decreased by 2.8 million with a change rate of −34%.

From 1990 to 2010, the total population and the elderly population in different regions increased, and the growth rate of the elderly was faster than the growth rate of the total population. In terms of the total population, there are some areas, including the RSHA, the type I counties in the RSHA, the type I counties in the RSHAS, the type II counties in the RSHA, and the type II counties in the RSHAS where the total population increased faster than the total national population growth rate. However, the growth rates of the total population in the type III counties in the RSHA and the RSHAS were slower than the national average. The contribution rates of the elderly to the total population in the RSHA and the type III counties in the RSHA and the RSHAS were higher than the national average. The number of children in the different regions decreased, and the rates of the decrease in the type I counties and the type III counties in the RSHA and the RSHAS were faster than others. The total population of the type I counties in the RSHA and the RSHAS increased due to the increase in the outer population caused by urbanization. Therefore, with the development of disaster prevention and mitigation policies, it is also feasible to adjust the flexibility of the population policy in combination with the local population structure. That is, the population policy in the type III counties can be relaxed. Conversely, the population policy in the type I counties can be stricter.

The changes in the population exposure from 1990 to 2000 (the earlier decades) and from 2000 to 2010 (the later decades) were similar to those of 1990 to 2010. The total population increased in both the RSHA and the RSHAS in different periods. The growth rates of the total population in the RSHA and the RSHAS were lower than the national average in the previous decade. The growth rate of the total population in the RSHA was similar to the national average. In contrast, the growth rate in the RSHAS was higher than the national average in the later decade. Particularly, the growth rates of the total population of the type I counties in the RSHA and the RSHAS were larger than those in the types II and III counties of the RSHA and the RSHAS, which were also higher than those in the RSHA, the RSHAS and China in both periods. The number of elderly in both the RSHA and the RSHAS increased in the two periods, and the growth rates were similar to the national average elderly population growth rate. However, the number of children in both the RSHA and RSHAS reduced in the two periods, and the rate of change was similar to the national average child population growth rate.

**Figure 6:**
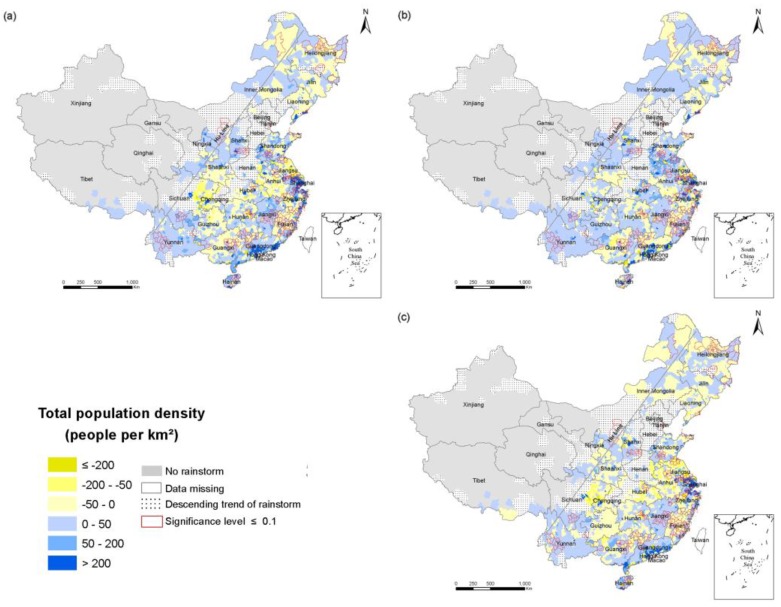
The change in the density of the total population in the RSHA (**a**) from 1900 to 2010; (**b**) from 1990 to 2000; (**c**) from 2000 to 2010.

**Figure 7:**
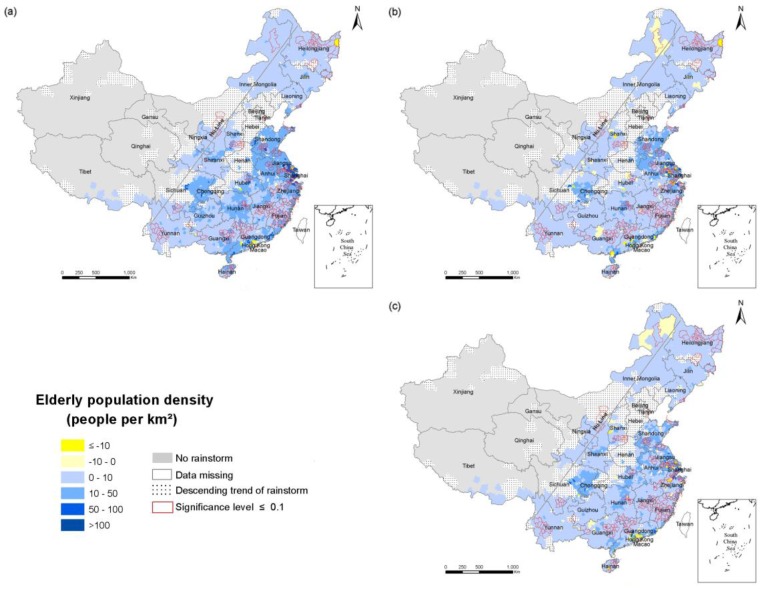
The change in the density of the elderly population in the RSHA (**a**) from 1900 to 2010; (**b**) from 1990 to 2000; (**c**) from 2000 to 2010.

**Figure 8:**
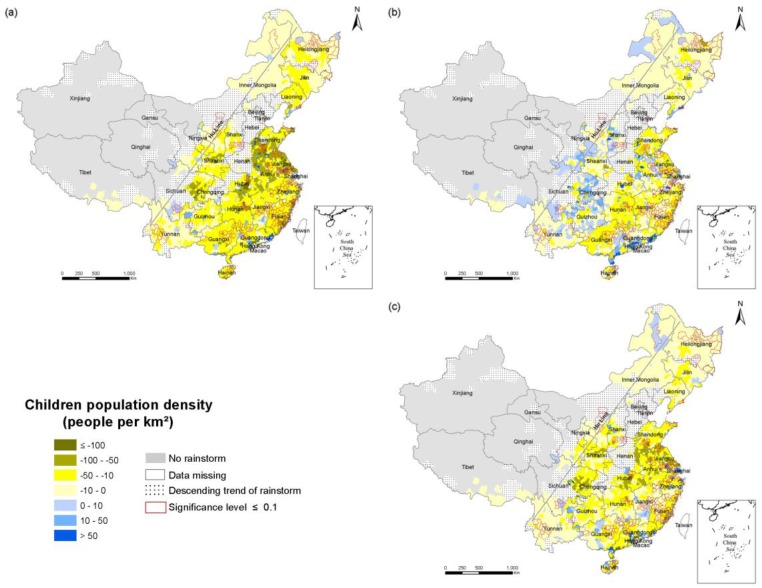
The change in the density of the child population in the RSHA (**a**) from 1900 to 2010; (**b**) from 1990 to 2000; (**c**) from 2000 to 2010.

To explore the temporal and spatial changes in the population exposure in the RSHA, maps of the change in density of the total population (Figure 6), the elderly (Figure 7) and children (Figure 8) in 1990–2010, 1990–2000 and 2000–2010 were developed.

From 1990 to 2010, the change of the total population exposure was heterogeneous, and the changes of the elderly and children exposures were regionally consistent in the RSHA. Specifically, the density of the elderly (children) of most counties in the RSHA increased (decreased) consistently. The highest values of the total population density increases were concentrated in Shanghai and Guangdong. The highest value of the elderly population density increase was concentrated in Shanghai. The highest values of the child population decrease were concentrated in Shandong, Jiangsu, Anhui, Hubei and Hunan. The areas of the RSHA where the total population density increased by more than 200 persons/km^2^ were mainly concentrated in Shandong, Shanghai, Zhejiang, Jiangsu, Guangdong. In terms of the elderly, from 1990 to 2000, the density of the elderly increased in the range of 10–50 persons/km^2^ in approximately half of the RSHA, and these areas were mostly concentrated in Shandong, Anhui, Yangtze River Delta, Hunan, Guangdong, Sichuan, and Jiangxi. The areas where the density of the elderly was more than 50 persons/km^2^ were mainly concentrated in Shanghai and Jiangsu.

## 4. Discussion

### 4.1. Predictions of the Future Population in the RSHA

There is considerable uncertainty surrounding the future level of population exposure due to the dynamic characteristics of population exposure. Considering the changes in China’s population policy, many researchers have predicted future demographic patterns according to different population conditions in China [41]. Both mortality and fertility have declined substantially; thus, population policies will determine the future population sizes and structures. When the population policy is fully transformed into a two-children-per-couple policy, the population will peak at 150 million in 2030. Aging is also a significant trend in China’s future demographic structure. In 2015–2035 and 2040–2050, the percentages of the elderly will reach 20% and 25%, respectively. The total number of elderly will reach 200 million in 2025 and 300 million in 2040. If the proportion of the population in the RSHA and the RSHAS in 2030 is similar to that in 2010, the total population in the RSHA and the RSHAS in 2030 will reach approximately 1000 million and 167 million, respectively. The elderly population in the RSHA and the RSHAS will reach 139 million and 22 million people, respectively.

The relevant departments in China need to pay close attention to the population exposure problem. In particular, the departments should pay close attention to the population exposure in the RSHA and the RSHAS. Because the risk of rainstorm-related disasters depends on the hazard, the exposure and vulnerability, and the increase of exposure level will undoubtedly increase the risk of disaster when other disaster conditions remain unchanged. Even if both the annual rainstorms and the population exposure levels increase, the relevant departments can also reduce the risks. By taking some simple measures to include older people in rainstorm-related disaster preparedness and response planning and management policy, the impact of rainstorm-related disasters on older people’s lives can be significantly reduced. Robust disaster plans which reflect the needs and capacities of older people before, during and after the disaster can mean fewer casualties and injuries, and help the older people to protect their assets, allowing for quicker and less costly response and recovery operations. For example, providing evacuation assistance, especially one-to-one assistance to older people can effectively reduce the casualties and injuries of the older people during a disaster. These measures could also strengthen monitoring and forecasting; provide timely early warning; educate and popularize disaster prevention knowledge, understanding of the older people’s risk perception and evacuation needs before the rainstorm disaster; provide medical assistance during the disaster; and offer long-term health and mental health care and economic help after the disaster.

### 4.2. Limitations

In this paper, we studied the historical population exposure and its changes based on the county units in the RSHA. In the future, the temporal resolution of the population exposure can be explored by examining simple yearly averages or nighttime and daytime distribution (and finer scales) frames. Second, the study of the population exposure in the RSHA is related to hazard, exposure and vulnerability aspects. The current study only focuses on the population exposure in detail and does not investigate the other two aspects. In terms of hazards, this paper identified the RSHA from daily precipitation data between 1975 and 1915 from meteorological stations. However, there is a certain degree of uncertainty because of the disorder of the weather system itself, the inaccuracies of the temporal and spatial scales of the weather system and the spatial interpolation method errors. In terms of the vulnerability of the elements at risk, there are many factors that should be considered, such as economic income, housing conditions, physical conditions, mental status and other external and internal factors. This paper only considered the physiological status of the population and analyzed the population exposure of the vulnerable elderly and children. The population exposure of different vulnerable groups needs more in-depth research. In addition, the analysis of the population exposure in the RSHA is semi-quantitative, and future research should study these trends quantitatively. Specifically, a hazards probability analysis and a study to identify the different vulnerable groups are needed. Future studies on population exposure to meteorological disasters should utilize high-precision model simulation methods to break away from the current method based on historical observations.

## 5. Conclusions

There is no doubt that the population of the 21st century is aging, and the world population is rapidly aging. Globally, the number of people 80 years and over will almost quadruple to 395 million by 2050 [42]. Due to the one-child policy and improved healthcare, the population in China as a whole is aging. The percentage of people over 65 in China rose from 63 million in 1990 to 119 million in 2010. By 2050, over one-quarter of the population of China will be over 60 years old [43].

China is one of the countries most affected by climate change and is facing negative impacts of climate change that include the challenges of increased climate-related disasters [44]. China also has the world’s largest population, and the largest elderly population. How can we determine if more or less of the vulnerable population will be exposed to extreme precipitation events? This paper analyzes the extreme precipitation in China from the background of global warming. This study also analyzes the changes in the total population and the vulnerable population as indicated by the elderly and children based on the population census data at the county level from 1990, 2000 and 2010 in China. The results of this study can provide a scientific reference for China to reduce the risks to the population of rainstorm-related disasters.

The study of the population exposure level in the RSHA shows that the total population and the elderly population in the types I, II and III counties increased from 1990 to 2010, and the child population decreased. The total population of the RSHA increased by more than 110 million at a rate of 13.8%, and the total population of the RSHAS increased by 20.6% to 25 million, which was higher than the increases in the RSHA and China (13.8% and 18.2%). The total population growth rate of the type I counties was fastest and increased by 28.6%. The elderly in the RSHA increased by 86.1% to 38.2 million, and the elderly in the RSHAS increased by approximately 6.1 million at a rate of 84.5%. The growth rate of the elderly is similar in all areas and is similar to the national rate of change (88.2%). The elderly in the RSHA accounted for 69.5% of the total national elderly population in 2010. The proportion of the increased aged population of the RSHA to that of China is 68.6% from 1990 to 2010. However, the total area of the RSHA only accounts for 52% of China’s mainland area.

The future total population and elderly population predictions based on China’s “two-child policy” indicates that the future population exposure of the RSHA and the RSHAS will undoubtedly increase. In addition, as we know, the risks of rainstorm disasters depend on three aspects, namely, hazards, vulnerability, and exposure or the elements at risk [45]. Therefore, the population risk will increase in the RSHA due to the high exposure level of the vulnerable population in the RSHA. Therefore, there is a need to call for more collective efforts to address the consequences of climate change and population aging, which are global issues that will be faced by many developed and developing countries. Something must be done to assist the policy-making and capacity building to help the aging population better adapt to climate change in the future, which will include developing bottom-up disaster prevention and mitigation strategies at the microscale. Moreover, the relevant departments of China need to take timely and effective measures to adjust their policies when the population exposure in the RSHA and the RSHAS reach a certain threshold.

## Figures and Tables

**Table 1:** Population exposure in the RSHA and RSHAS in 1990, 2000 and 2010, units: millions.

Population and Year		RSHA				RSHAS				Mainland China
		I	II	III	Total	I	II	III	Total	Total
Total population	1990	36.3 (3.2)	217.9 (19.2)	545.9 (48.1)	800.1 (70.6)	27.3 (2.4)	66 (5.8)	29.8 (2.6)	123.1 (10.9)	1133.7 (100)
	2000	40.7 (3.2)	234.1 (18.5)	591.4 (46.7)	866.2 (68.4)	30.7 (2.4)	69.6 (5.5)	32.7 (2.6)	133 (10.5)	1265.8 (100)
	2010	46.6 (3.5)	261.2 (19.5)	602.4 (45)	910.2 (67.9)	36.1 (2.7)	79.2 (5.9)	33.1 (2.5)	148.4 (11.1)	1339.7 (100)
Elderly population	1990	2 (3.2)	12.9 (20.4)	29.5 (46.7)	44.4 (70.3)	1.5 (2.3)	4.1 (6.5)	1.6 (2.5)	7.2 (11.3)	63.2 (100)
	2000	2.8 (3.2)	18.3 (20.7)	40.9 (46.4)	62 (70.3)	2.1 (2.4)	5.7 (6.4)	2.3 (2.6)	10 (11.4)	88.1 (100)
	2010	3.7 (3.1)	23.9 (20.1)	55 (46.3)	82.6 (69.5)	2.8 (2.3)	7.4 (6.3)	3 (2.5)	13.2 (11.1)	118.8 (100)
Children	1990	11.1 (3.5)	61.2 (19.5)	153.4 (48.8)	225.6 (71.9)	8.3 (2.6)	17 (5.4)	8.9 (2.8)	34.2 (10.9)	314 (100)
	2000	9.2 (3.2)	53.4 (18.4)	141.3 (48.7)	203.8 (70.3)	6.8 (2.4)	14.5 (5)	8 (2.8)	29.4 (10.1)	289.8 (100)
	2010	7.2 (3.3)	45.1 (20.3)	103.3 (46.4)	155.7 (70)	5.5 (2.5)	12.4 (5.6)	5.9 (2.7)	23.7 (10.7)	222.5 (100)

Note: the numbers in the brackets are the percentages of national total population, national elderly population and national child population, unit: %; RSHA: rainstorm risk hazard area; RSHAS: the risk hazard area of rainstorm with significant linear regression effect.

**Table 2:** The proportions of the total population that are vulnerable in different regions and years, unit: %.

Area	1990		2000		2010	
	Percentage of the Elderly	Percentage of Children	Percentage of the Elderly	Percentage of Children	Percentage of the Elderly	Percentage of Children
RSHA	5.6	28.2	7.2	23.5	9.1	17.1
RSHAS	5.8	27.8	7.5	22.1	8.9	16
Mainland China	5.6	27.7	7.0	22.9	8.9	16.6

**Table 3:** The change of population exposure, unit: millions.

Area		RSHA				RSHAS				Mainland China
		I	II	III	Total	I	II	III	Total	Total
From 1990 to 2010	Total population	10.4 (28.6)	43.3 (19.8)	56.6 (10.4)	110.2 (13.8)	8.8 (32.2)	13.2 (20)	3.3 (11)	25.3 (20.6)	206 (18.2)
	Elderly population	1.7 (84.4)	11 (85.6)	25.5 (86.4)	38.2 (86.1)	1.3 (87.4)	3.4 (82.1)	1.4 (88)	6.1 (84.5)	55.7 (88.2)
	Children	−3.9 (−34.7)	−16.1 (−26.3)	−50 (−32.6)	−70 (−31)	−2.8 (−34.2)	−4.6 (−27.2)	−3 (−33.7)	−10.5 (−30.6)	−91.5 (−29.1)
From 2000 to 2010	Total population	5.9 (14.5)	27.1 (11.6)	11 (1.9)	44 (5.1)	5.4 (17.6)	9.6 (13.8)	0.4 (1.2)	15.4 (11.6)	73.9 (5.8)
	Elderly population	0.8 (29.1)	5.7 (31.1)	14.2 (34.7)	20.7 (33.4)	0.7 (32)	1.8 (31.8)	0.7 (32.8)	3.2 (32.1)	30.7 (34.9)
	Children	−2 (−21.4)	−8.3 (−15.5)	−37.9 (−26.9)	−48.2 (−23.6)	−1.4 (−19.9)	−2.1 (−14.7)	−2.1 (−26.6)	−5.6 (−19.2)	−67.3 (−23.2)
From 1990 to 2000	Total population	4.5 (12.3)	16.1 (7.4)	45.6 (8.3)	66.2 (8.3)	3.4 (12.4)	3.6 (5.5)	2.9 (9.7)	9.9 (8)	132.1 (11.7)
	Elderly population	0.9 (42.9)	5.4 (41.5)	11.4 (38.4)	17.6 (39.5)	0.6 (42)	1.6 (38.1)	0.7 (41.6)	2.8 (39.7)	25 (39.5)
	Children	−1.9 (−16.9)	−7.8 (−12.8)	−12.1 (−7.9)	−21.8 (−9.7)	−1.5 (−17.8)	−2.5 (−14.7)	−0.9 (−9.6)	−4.8 (−14.1)	−24.2 (−7.7)

Note: The numbers in the bracket are the percentage of change that is calculated by (P*_i_* – P*_j_*)/P*_j_* × 100%, *i*, *j* = {2010, 2000, 1990}, *i* > *j*, unit: %.
